# Long-term follow-up of a community sample of adolescents with frequent headaches

**DOI:** 10.1186/s10194-018-0908-5

**Published:** 2018-09-04

**Authors:** Bo Larsson, Johannes Foss Sigurdson, Anne Mari Sund

**Affiliations:** Regional Center for Child and Youth Mental Health and Child Welfare – Central Norway, NTNU, Klostergat. 46/48, N-7489 Trondheim, Norway

**Keywords:** Adolescence, Headache, Prevalence, Incidence, Long-term follow-up

## Abstract

**Background:**

Several outcome studies have reported on the short- and long-term effects of migraine in selected clinical samples of children and adolescents. However, current knowledge of the course, incidence, and outcome predictors of frequent headaches in early adolescents in community populations is limited, and little is known about the long-term effects. Headache remains untreated in most of these young people. Here we examined the course, incidence, and outcome predictors of frequent headaches (at least once a week) over the long term (14 years) using previously assessed data at the baseline and 1-year follow-up of early adolescents.

**Methods:**

Out of an original sample of 2440 who participated in the first two assessments, a sample of 1266 participants (51.9% response rate) aged 26–28 years (mean = 27.2 years) completed an electronic questionnaire comprising questions about their headache frequency and duration at the long-term follow-up. These headache characteristics together with gender, age, parental divorce, number of friends, school absence, impairment of leisure-time activities and seeing friends, pain comorbidity, and emotional (in particular, depressive symptoms) and behavioral problems were analyzed.

**Results:**

In these young people, 8.4% reported frequent headaches (at least once a week) at the extended follow-up, while 19% of the participants having such headaches at baseline again reported such levels with a negligible gender difference. Over the follow-up period, 7.4% had developed frequent headaches, and a higher percentage of females reported such headaches (11.3% in females, 1.5% in males). In a multivariate model, frequent headaches at the baseline, gender (worse prognosis in females), impairment of leisure-time activities and seeing friends, and higher level of depressive symptoms significantly predicted headache frequency at the long-term follow-up.

**Conclusions:**

Our findings suggest that gender, greater social impairment, and comorbid depressive symptoms are important indicators for both the short- and long-term prognosis of frequent headaches in early adolescents in community populations.

## Background

Headache is one of the most common health and somatic complaints reported by adolescents in the general population [1–5]. In two reviews of epidemiological surveys conducted in various countries and cultures, the estimated mean prevalence rates of unspecified headaches among children and adolescents were 54.4% [4] and 58.4% [4, 5]. A striking increase in the prevalence of unspecified headaches and migraine occurs at the onset of puberty among girls but not boys [4–8]. Such complaints can negatively affect the quality of life and lead to impairments in daily functioning such as school performance, absence and recreational activities [9]. Severe or frequent headaches in children and adolescents are also related to emotional problems; in particular, anxiety, depression, behavioral problems [10, 11], other somatic symptoms, social impairment, problems interacting with peers, pain comorbidity, and parental headache [12, 13].

The persistence of headaches among children and adolescents experiencing unspecified headaches or migraine is an important aspect and burden of these complaints. Several retrospective and prospective follow-up studies of clinical samples of children and adolescents experiencing primarily migraine have reported on both the short-term and longer perspectives [14–17]. However, such samples typically include young people with more complicated neurological symptoms, frequent attacks, or impairment compared with those who are not referred [18].

In community surveys conducted in Scandinavia and Germany, the course of unspecified headaches in these age groups has been investigated primarily over the short term covering 1–3-year periods [14, 19–25] for which the overall persistence of headaches in the general population has been found to be high. For example, at the 1-year follow-up evaluation, slightly more than half (57%) of children and adolescents with headaches still experienced such complaints, as reported by parents [19]. In another similar longitudinal study, one-third of early adolescents reported frequent headaches (at least once a week) [20]. In a 3-year follow-up of school children, most of those with headaches (80%) still reported headaches, and female gender and the frequency of headaches predicted the persistence of these complaints [22].

To date, the existing information on the long-term prognosis of headaches in children and adolescents in community populations is limited. In a pioneering survey conducted in the 1950s, Bille followed a subsample of school-aged children with non migrainous headaches for a 6-year period [26] and another group with pronounced migraine at various time points for an extended period up to 40 years [27, 28]. Although about half of the children with migraine still had attacks around the age of 50 years, the prognosis was better for men than for women. In a school-based sample, Özge and collaborators followed 1155 children for a 6-year period up to mid-adolescence [29]. The overall headache prevalence increased from 45.2 to 78.7%, and most students had stable headaches, although the headache diagnoses showed a high transition rate over time. In this sample, female gender and having a parent with headaches predicted headache stability. In a 5- and 10-year follow-up of 8-year old children in a community study, Schmidt and colleagues found a high stability of headaches across the two reassessment points up to late adolescence (73 and 47%, respectively) [30].

In a 13-year follow-up of a sample of 335 school children into young adulthood (21–27 years of age), Brattberg [31] reported that about one-quarter still had headaches, while the same percentage represented incident cases. A headache frequency of at least once a week predicted the persistence of headaches. A longitudinal 15-year study of headaches severe enough to disturb daily life in the past 6 months collected data for children starting school (age 7 years) and again at the ages of 13–14 and 22 years [32]. The overall headache prevalence was virtually unchanged during and after puberty in this study. However, these estimates were surprisingly low, probably because of the conservative definition of recurrent headaches associated with impairment used in the study. In a general population study of migraine and other headaches among children starting school from the same research group in Turku, Finland, Anttila and collaborators examined long-term trends at two separate time points in adulthood when the participants were 22 and 35 years of age in separate cohorts [33]. They found an increase in the incidence of frequent headache and migraine in both boys and girls over a 30-year period. In another extensive longitudinal study of a large sample (*N* = 11,407) from the general population in the UK, parent-reported headaches among children starting school were associated with the frequency of headaches, multiple somatic symptoms, and psychiatric morbidity at the age of 33 years [8].

We have previously reported on the incidence, course, and 1-year outcomes of frequent headaches among early adolescents in a representative sample of the general population [19].We also assessed the influence of several potential predictors of outcomes: gender, age, relationships with peers, parental divorce, pain comorbidity and impairment related to disease or pain, and behavioral and emotional problems; in particular, depressive symptoms. In the present study, we investigated these aspects further in a long-term (14-year) study of the participants in our original sample.

## Methods

### Study design

The Youth and Mental Health Study is a longitudinal study conducted in Mid-Norway that assesses risk and protective factors in the development of mental health in adolescents aged 12–15 years [34]. In a first phase in 1998, a representative sample of 2813 students (98.5% attending public schools) from 22 schools in two counties of Mid-Norway (South and North Trøndelag), which included urban and rural areas, was drawn with a probability according to size (proportional allocation) from a total population of 9292 children.

### Sample and assessment points

Baseline data (T1) were collected from 2464 adolescents with an 88.3% response rate and a mean age of 13.7 years (SD 0.58, range 12.5–15.7); 50.8% were girls. One year later (T2), 2432 respondents were reassessed at a mean age of 14.9 years (SD 0.6, range 13.7–17.0; 50.4% girls). Other details of the sample selection are presented elsewhere [31].

**Fig. 1:**
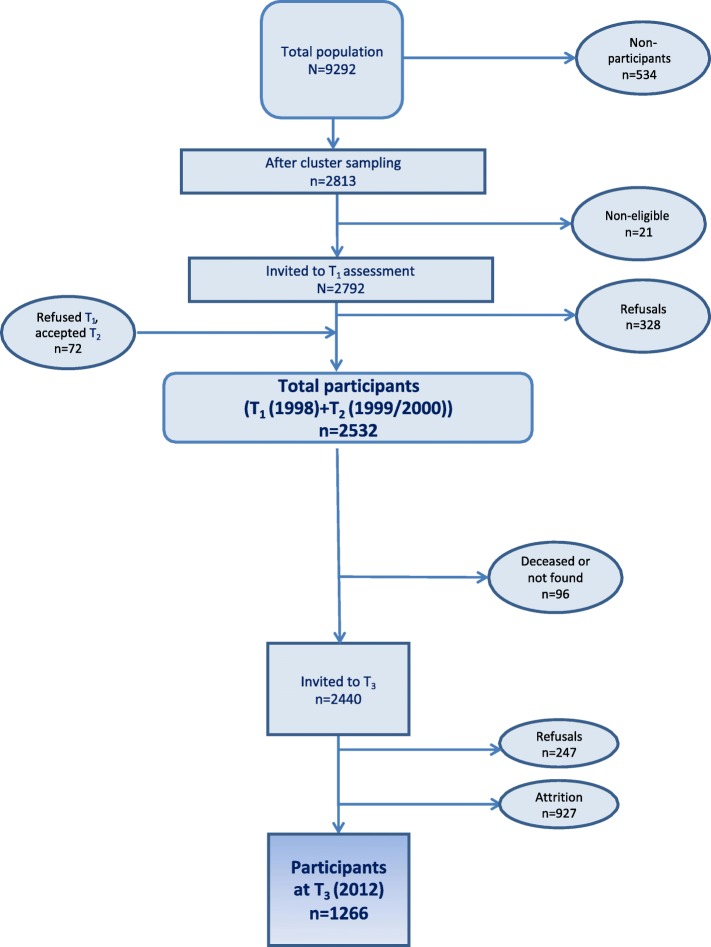
Participant flow in the Youth and Mental Health Study

Individuals participating at T1 or T2 (*N* = 2532) were again asked to participate in a long-term follow-up study in young adulthood during the spring of 2012 (T3) about 14 years after T1 when they were a mean age of 27.2 years (SD 0.59, range 26.0–28.2). At this time, 92 of the participants were not eligible because of death (*n* = 13) or no identifiable home address (*n* = 79). Thus, 2440 young adults were invited to participate in this follow-up study, of which 1266 (51.9%) participated (56.7% females) (see Fig. 1 for details of participant flow). The headache frequency at the baseline did not differ significantly between participants and nonparticipants at T3.

In the first two waves, data were collected through questionnaires completed during two school hours. In the long-term follow-up study in adulthood, all assessment data were collected electronically.

### Assessment measures

#### Background factors and predictors

In addition to gender and age, the following psychosocial factors were included because they were identified by an extensive review of risk factors as being important predictors in children and adolescents experiencing migraine or tension-type headaches referred to a clinic compared with headache-free controls [35]: *parental divorce, low number of friends (more common among children with tension-type headaches), and school absence (more common among children with migraine* [9]*.* Adolescents had provided information about parental divorce before T1. The number of friends at the assessment point was subgrouped into “0 to 1” and “2 or more friends” categories. At T1, 27.7% (*N* = 684) of the adolescents had divorced parents, and 4% of the students reported having a low number of friends (0 to 1).

#### Impairment

At T1, the adolescents were asked whether they had been absent from school because of disease, injury, or pain during the past 12 months. The possible answers were “No,” “A few days,” “1–3 weeks,” “1–3 months,” and “More than 3 months”. They were also asked whether they had decreased or stopped leisure-time activities or seeing friends because of disease, injury, or pain during the past 12 months (“No” or “Yes”).

Because various types of internalizing problems, such as anxiety, depression, somatic symptoms, and behavioral problems are often associated with recurrent headaches in children and adolescents, these domains were included as baseline predictors in our previous 1-year follow-up study [20] as well as in the present long-term follow-up evaluation.

#### Youth self-report

To provide a broad assessment of the adolescents’ emotional and behavioral problems, a Norwegian version of the widely used and standardized Youth Self-Report (YSR) problem scale was used [36]. The instrument includes 103 items and 16 socially desirable items. The subject was asked to rate each item on a three-point scale (0 = “Not true”; 1 = “Somewhat or sometimes true”; and 2 = “Very true or often true”) for the past 6 months. In addition to a total problem score, two broadband dimensions—internalizing and externalizing syndromes—can be formed from the subscales. The internalizing syndrome comprises three narrow-band syndromes—Withdrawn, Somatic complaints, and Anxious/depressed—and the externalizing syndrome comprises Aggression and Delinquent subscales. All of these subscales were included in this study. Three pain items (aches or pains, headaches, stomachaches) in the somatic subscale were excluded from the sum score.

#### The mood and feelings questionnaire

To assess specifically depressive symptoms among the adolescents using a comprehensive and standardized measure, the Mood and Feelings Questionnaire (MFQ) was completed by participants [37]. This is a 34-item questionnaire designed for children and adolescents aged 8–18 years to report depressive symptoms as specified by the DSM-III-R diagnostic system. The individual is asked to report on his or her feelings during the preceding 2 weeks. Responses are made to statements on a 3-point scale (“Not true,” “Sometimes true,” and “True”), and total scores range from 0 to 68. The MFQ has been shown to have high internal consistency, test–retest stability, and convergent validity in the present research project [34].

#### Headache and other pain

On all three test occasions (T1–T3), the adolescents/young adults were asked whether they had regular and troublesome pain complaints (except for menstrual pain). If they answered positively, they were asked to rate the frequency of pain (“1–3 times a month,” “1–3 times a week,” or “Daily or almost daily”) for headache, stomach pains, back pains, and pains in the arm or leg (hereafter called limb pain). They were also asked about the duration of pain (“Less than an hour,” “1–4 h,” and “More than 4 hours”). Those who reported at least weekly pains were regarded as experiencing frequent pain, a criterion also used here as our prime outcome variable in the long-term follow-up evaluation at T3.

### Statistical analyses

Descriptive statistics included percentage, mean, and standard deviation (SD) for continuous variables. The chi-square test was used to analyze bivariate correlations between categorical variables. Analyses of percentages were conducted using the z-distribution. Student’s *t*-test was used to examine differences in means between independent groups for continuous variables. To examine the relative importance of significant explanatory factors in bivariate analyses, logistic regression analyses using the enter method were performed. In all analyses, *p <* .05 was considered to be significant.

## Results

### Headache prevalence

At the long-term follow-up and ages 26–28 years (T3), 82.5% of the participants reported having headaches rarely or not at all, 9% had headaches 1–3 times a month, 6% had headaches 1–3 times per week, and 2.4% had headaches occurring daily or almost daily. Thus, 8.4% of participants reported frequent headaches at least once a week. Of the participants who experienced headaches, 43.3% reported a duration of 1–4 h, 29% had a shorter duration, and 27.6% had a longer duration. The association between headache frequency and duration was significant and was strongest for those with the longest duration and headaches occurring daily or almost daily (6.9%), [χ^2^ (4) = 16.14, *p <* .001].

The association between gender and headache frequency was significant, [χ^2^ (3) = 46.55, *p <* .001] females reported a higher frequency than males; in particular, for frequent headaches (12.7% versus 2.8%, respectively). Similarly, headache duration was significantly related to gender, [χ^2^ (2) = 15.53, *p <* .001]. Gender was not significantly associated with the longest duration, but about half of the females (51.2%) experienced headaches lasting 1–4 h, whereas only about one-quarter of the males (23.5%) experienced headaches of this duration.

### Pain comorbidity

At the long-term follow-up, 6.6% of participants reported frequent headaches only, whereas 1.8% of the participants with such headaches also had another frequent comorbid pain (in the stomach, back, or limbs: 14, 5.6 and 1.9%, respectively). This was significantly higher than for those with no such headache, [χ^2^ (1) = 8.11, *p <* .001].

### Course and incidence of headaches

Of those participants who had frequent weekly headaches at T1, 19.1% also reported such headaches at the long-term follow-up. Having weekly headaches at T1 or T2 or both occasions was significantly associated with reporting frequent headaches at T3, [χ^2^ (3) = 26.53, *p <* .001] as compared with not having weekly headaches at T1 (17.1 and 6.7%, respectively). A slightly but not significantly higher stability between T1 and T3 was found for females than for males with frequent headaches (18.1 and 15.7%, respectively). Headache duration at T1 did not predict duration or frequency of headaches at T3, and age at T1 was not significantly related to the occurrence of headaches at T3.

At the long-term follow-up, 7.4% of participants not having frequent headaches at T1 reported frequent headaches (incident cases). The difference in incidence rates between gender was highly significant, (Z = 6.81, *p* < .001): 11.3% of the females but only 1.5% of the males had reported frequent headaches at T3.

### Predictors of long-term outcome

The bivariate analyses showed that, besides gender (see above), parental divorce, pain comorbidity at T1, and impairment during leisure-time activities or seeing friends because of disease, injury, or pain were significantly associated with reports of frequent versus infrequent versus headaches at T3, [χ^2^ (1) = 11.32, *p <* .001, χ^2^ (1) = 8.11, *p <* .001, and χ^2^ (1) = 5.13, *p <* .05, respectively]. The percentages reporting frequent versus infrequent headaches at T3 for each variable were as follows: for parental divorce, affirmed vs not: 13.1% vs 7.0%; for pain comorbidity at T1, affirmed vs not: 14.4% vs 7.6%; and impairment during leisure-time activities or seeing friends because of disease, injury, or pain, affirmed vs not: 13.9% vs 7.6%, respectively). However, the number of friends and school absence were not significantly related to long-term outcome. Further analyses showed that all internalizing problem subscales on the YSR and the total mean scores on the MFQ significantly predicted outcome—reporting frequent or infrequent headaches at T3. However, the mean total values for the two externalizing subscales (aggressive and delinquent behavior) at T1 did not predict headache frequency at T3.

### Multivariate analysis

The significant explanatory factors in our bivariate analyses (except for the anxious/depressed subscale on the YSR, which was excluded because of the high correlation with the MFQ sum score; *r* = .68) were entered into a logistic regression analysis to examine their relative importance. The results (*n* = 1087) showed that the following explanatory baseline factors significantly predicted frequent headache (vs infrequent) at T3: gender (B = 1.64, SE = 0.34, *p* < .001, odds ratio (OR) = 5.14, 95% confidence interval (CI) = 2.65, 9.95); frequent headaches (B = 0.74, SE = 0.32, *p* < .05, OR = 2.10, 95% CI = 1.12, 3.94); impairment of leisure-time activities and seeing friends (B = 0.80, SE = 0.33, *p* < .05, OR = 2.22, 95% CI = 1.17, 4.20); and sum scores of depressive symptoms on the MFQ (B = 0.22, SE = 0.11, *p* < .05, OR = 1.02, 95% CI = 1.00, 1.05). The Hosmer–Lemeshow test was nonsignificant, which indicated a goodness of fit for the model.

## Discussion

In the present long-term follow-up study, early adolescents (aged 12–15 years) in a representative general population sample were reassessed about 14 years later in adulthood (mean age 27.2 years) with a focus on the prevalence, incidence, course, and outcome predictors of frequent versus infrequent headaches.

Our prevalence estimates for these young adults and a report of headaches at least once a month at the extended follow-up was low—17.5%—and only 8.4% of participants reported frequent headaches occurring once a week or more. These relatively low estimates of headache frequency may reflect our introductory phrasing of the question, “Do you suffer from regular pain?” This item was used to allow comparisons of prevalence estimates and changes across the follow-up period with the rates reported by early adolescents at the baseline and our previous 1-year follow-up evaluation [20]. The present prevalence estimates are similar to those of a large epidemiological survey of adults conducted in one of the counties and the same health region as included in the present baseline study. In the previous study, 11.6% of females and 4.4% of males reported frequent headaches (“Have you suffered from headaches during the past year?”) [38]. Furthermore, our estimate of daily or almost daily headaches (2.4%) was identical to that reported in the previous survey of adults. Although this latter survey was conducted some years before our present follow-up evaluation, our estimates seem to be reliable and are likely to reflect the true prevalence rates of headaches in this age group of young adults.

Over a short-term perspective of 1–3 years, the persistence of headaches has been shown to be high; that is, between 33% and nearly 80% of children and adolescents still experienced headaches at reassessment; in particular, frequent headaches [19, 20, 22, 23]. High persistence rates have also been reported in follow-up studies for longer periods (5–10 years) in which half to most (47–83.5%) of the school children still had headaches in adolescence [29, 30], whereas about one-quarter of young adults still reported headaches in longer follow-up evaluations (13–15 years) [31, 32]. To date, the longest follow-up studies of school-aged children were performed by Bille, who examined the course of pronounced migraine over 30–40-year intervals when about 50% of the participants were about 50 years of age and still experienced migraines [28].

In the present study of early adolescents with frequent headaches, who were followed for about 14 years, 19% of the participants reported such headaches, but there was a negligible gender difference. Our findings seem to be similar to those of Aromaa and colleagues, who performed a 15-year follow-up study of frequent headaches in school children as they became young adults [32]. However, their definition of frequent headaches during the past 6 months also included impairment of the child and specific areas in daily life, which may have contributed to very low prevalence rates and smaller numbers in subgroups.

While annual incidence rates of headaches were reported for children and early adolescents in two community-based surveys [19, 20], our previous study estimated the 1-year incidence for frequent headaches as 6.5%. In a reassessment of school children with no headache at baseline, Özge and colleagues [29] reported that three-quarters had developed some form of headache disorder at the 6-year follow-up. In longer follow-up studies of 14 and 26 years, 25.1% [8] and 12.2% [29] of school children had developed unspecified headaches, respectively. In a 16-year follow-up of children in a headache-free control group, 11% had developed migraine [28]. However, we found no extended follow-up studies using the same criterion for frequent headaches as used in the present study, in which 7.4% of participants had developed frequent headaches over the 14-year period. Importantly, we found a strong gender difference: females had a higher risk than males (11.3 and 1.5%, respectively) for developing frequent headaches in adulthood. A previous epidemiological survey conducted in the same health region [38] reported a similar gender difference in prevalence rates of frequent headaches (at least six episodes per month) among 20–29-years olds: 11.6 and 4.4% for females and males, respectively.

The predictors of outcomes have been examined further in long-term follow-up studies. For example, a 6-year follow-up of school children into adolescence in Turkey studies the predictors of incident cases and persistence of headache [29]. In that study, having a sibling with headache and a working mother increased the risk for developing headaches in adolescence for headache-free children, whereas having a mother with headache and female gender were associated with persistence of headaches. In the 15-year follow-up by the Turku, Finland, research group, headaches at school entry also predicted headache and migraine in early adulthood [32]. In the longer 26-year follow-up of children starting school in the UK, frequent headaches predicted headaches, multiple physical symptoms, and mental morbidity in adulthood [8].

In their extensive review of the potential risk factors that may differentiate between various headache disorders in cross-sectional clinical and school-based samples, Karwautz and collaborators [35] reported that parental divorce, low number of friends, and school absence were important discriminators between headache disorders such as migraine and tension-type headaches compared with headache-free controls. A previous study of adolescents in a community sample also found a strong relation between pubertal development and an increase in headaches and depressive symptoms in girls, which suggested a possible link to hormonal changes during puberty in girls [39].

Whether these potential indicators found in cross-sectional studies also predict short- and long-term outcomes in prospective outcome studies of children and adolescents with recurrent headaches in community-based studies is an important issue. We evaluated the importance of these short-term predictors and others in our previous 1-year follow-up study of early adolescents in the original sample [20]. In that study, unspecified frequent headaches at the baseline, gender (worse prognosis for girls), impairment of leisure-time activities and seeing friends, and high levels of depressive symptoms contributed significantly to the outcome. Of particular note is that the same set of baseline factors also predicted the frequency of headaches in the present 14-year follow-up evaluation of some of the original sample. This established model shows that the frequency of headaches, presence of comorbid depressive symptoms, and social impairment are important predictors of the short- and long-term prognosis for frequent headaches in early adolescents in unselected community samples.

The reasons for these variables having such a significant effect both in short term and long-term perspectives on frequent headaches are likely to be linked to relationships between vulnerability and resilience factors in adolescence. Increasing demands both developmentally, socially and academically might produce a stress pattern influencing the development and persistence of frequent headaches in youth. They may also lead to depressive symptoms, and their poor regulation often co-occuring with poor social skills and having few friends and avoidance of social activities, contribute to a negative and accumulative spiral into adulthood.

We note that many of these young people with such headaches do not seek or receive clinical treatment but rather learn to endure their headaches without complaint. One important factor increasing the likelihood that adolescents with frequent headaches also seek clinical treatment is degree of social disability as reflected by levels on a standardized measure such as the PedMIDAS [40] developed to assess social functioning among children and adolescents with migraine in a tertiary clinical sample. Here frequency of headaches was positively correlated to levels of disability, but less so for duration. While substantially lower disability levels have been observed in community samples of children and adolescents with frequent headaches [41, 42], those with a chronic migraine disorder have reported significantly higher disability levels.

### Study limitations and strengths

Our study has some limitations. First, although the response rates at the long-term follow-up did not differ between adolescents with frequent headaches and those who were headache free at the baseline, the response rate after 14 years was fairly low (51.9%). Because of our assessment procedures and the use of self-report in the questionnaires and electronic responses, no formal headache diagnoses were established. Our definition of headache and other pain included “troublesome” complaints, which may have lowered the prevalence estimates in the present study. The reported data in the present study were part of an extensive long-term follow-up evaluation of adolescents and did not specifically focus on headache aspects only. Although our findings suggest a strong rate of persistence across the long-term follow-up period, we cannot rule out natural and spontaneous fluctuations in the rate of frequent headaches during the extended follow-up from adolescence into adulthood. Noteworthy here is that our predictive model at the 1-year follow-up was almost identical to the one obtained for our present long-term follow-up; however, our findings are likely to be restricted to the outcome for adolescents with frequent headaches in community samples. Whether it is also valid for adolescents with more complicated and frequent headaches in clinical samples should be confirmed.

Major strengths of the present study are the large sample, the long-term follow-up, and the inclusion of adolescents from various geographic (urban and rural) areas in the baseline sample. The assessment of headache was also based on the participants’ current experience without the need for recall, which therefore minimized the potential for memory bias. The same predictive multivariate model of frequent headaches as obtained at the 1-year follow-up was reestablished in our long-term follow-up evaluation, which strengthens the validity of the present findings.

## Conclusions

We found that a sizable proportion of adults aged 26–28 years reported frequent headaches to the same extent as they did in early adolescence. This and other findings of short- and long-term follow-up studies of community samples of children and adolescents suggest that there is strong risk for continuation of frequent headaches commonly associated with higher levels of emotional problems and impairment in social activities. In the long-term perspective, they are likely to have developed into chronic tension-type or migraine headaches or a combination. Although the prevalence of headaches improves in a substantial proportion of adolescents as they move into adulthood, the persistence of frequent headaches, particularly among girls, along with higher levels of depressive symptoms and impairment, emphasizes the need to provide effective psychological and pharmacological treatments to reduce frequent headache complaints and their associated social burden.

In future longitudinal research, more frequent and repeated assessment over time will reveal whether changes in persistence, improvement rates, and relapses occur in adolescents with recurrent headaches. This information will improve the identification of individuals experiencing persistent frequent headaches over extended periods. More importantly, for these people, the potential influence of gender, presence of depressive symptoms, and extent of impairment should be tested in the context of controlled treatment trials to examine whether these factors also contribute to changing the outcome.

## Abbreviations

B: Unstandardized B coefficient
CI_95%_: Confidence interval
OR: Odds ratio
SE: Standard error
T1-T3: Time points
